# Mechanical and microstructural properties of iron mining tailings stabilized with alkali-activated binder produced from agro-industrial wastes

**DOI:** 10.1038/s41598-023-42999-x

**Published:** 2023-09-21

**Authors:** William Mateus Kubiaki Levandoski, Suéllen Tonatto Ferrazzo, Giovani Jordi Bruschi, Nilo Cesar Consoli, Eduardo Pavan Korf

**Affiliations:** 1 Graduate Program in Environmental Science and Technology, Universidade Federal da Fronteira Sul, Erechim, RS 99700-970 Brazil; 2 Graduate Program in Civil Engineering, Universidade Federal do Rio Grande do Sul, Porto Alegre, RS 90035-190 Brazil

**Keywords:** Environmental impact, Civil engineering

## Abstract

This study evaluated the stabilization of iron ore tailings (IOTs) with an alkali-activated binder (AAB) produced from sugar cane bagasse ash, hydrated eggshell lime, and sodium hydroxide solution. Unconfined compressive strength, split tensile strength, initial shear stiffness, mineralogy, chemical composition, and microstructure of IOTs-AAB were evaluated. Strength values up to 6.59 MPa were achieved after 28 days-curing at 40 °C. Reducing porosity and increasing the binder content improved the overall mechanical behavior. N-A-S-H like gels were identified in IOTs-AAB mixtures. Finally, longer curing times led to more compact structures.

## Introduction

The world reserves of iron ore are around 170 billion tons. More than 2.4 billion tons of iron ore were processed in 2019, in which 36.7% correspond to the world’s largest producer, Australia, followed by Brazil with 18.9%^1^. However, the activities of the mining sector generate several negative impacts (e.g., depreciation of surface water quality, vegetation suppression, intervention in permanent preservation areas, atmospheric pollution, and landscape alteration)^2,3^. In addition to these impacts, low-frequency events such as tailing dam failures may result in devastating effects, such as the ones observed in the failures of Mariana and Brumadinho in the years of 2015 and 2019, respectively^4,5^. Furthermore, the processing and transportation of minerals include activities that generate high amounts of greenhouse gases^6–8^ and a large volume of waste. Globally 1.4 billion tons/year of iron ore tailings (IOTs) are generated, mainly in Australia, China, and Brazil^9^. In Brazil, around 260–275 million tons of waste are generated and stored in tailing dams each year^10,11^. Considering the high generation of mining tailings, methods that may reduce the associated risks while even monetizing these materials are growing in interest. Among these techniques, chemical stabilization is being presented as an interesting alternative^12^.

Portland cement has been extensively studied on the stabilization of mining tailings, being known for improving the materials mechanical properties^13–15^, heavy metal immobilization^16^, and acid neutralization^17^. However, the production of conventional Portland cement demands intense consumption of energy and natural resources, while emitting a significant amount of air pollutants^12^. It is estimated that the production of 1 ton of Portland cement requires 1.5 tons of raw materials (70% limestone) and generates 0.94 tons of carbon dioxide, which corresponds to 7% of the world greenhouse gas emissions^18^.

As an alternative to Portland cement, waste-like materials rich in aluminosilicates are being used as precursors for the production of alkali-activated binders (or alkaline types of cement)^19^. The alkali-activation technique can be defined as the reaction between a source of aluminosilicates under alkaline conditions^20^, resulting in the formation of C-A-S-H (hydrated calcium aluminosilicate) or N-A-S-H (hydrated sodium aluminosilicate) gels and structures^21^. The most exploited industrial by-products are still metakaolin, blast furnace slag, steel slag and fly ash^21–24^. Nevertheless, other sources of aluminosilicates, such as mining waste^12^, ceramic powder^25^, glass waste^26,27^ and rice husk ash^28^ have also been used for the production of alkali-activated binders.

Different precursor materials for alkali-activation can also be presented in the form of sugar cane bagasse ash and eggshell: two agro-industrial wastes with no added value and generally disposed in landfills or inappropriate areas^29^. Brazil is the world’s largest producer of sugarcane, with an estimated production of 579 million tons in 2022^30^. Considering that 1 ton of processed sugarcane (sugar and ethanol production) generates 300 kg of bagasse^31^, and that the burning (electricity generation) of 1 ton of this by-product results in 24 kg of ash^32^, it can be inferred an annual generation of 80 thousand tons of waste in the country. This ash is considered a pozzolanic material, with silica, aluminum and iron oxide contents greater than 70%^33^. On the other hand, eggshell waste is a residue produced from domestic and industrial consumption of eggs. Around 65.5 million tons of eggs are produced annually in the world, 45% by China, while Brazil is responsible for 3.4%^34^. Considering that eggshells represent 11% of the total mass of the egg^35^, a worldwide production of 7.2 million tons/year can be inferred. This waste corresponds to a rich source of calcium carbonate (CaCO_3_) and its recycling, through calcination, creates a popularly product known as eggshell lime (CaOH_2_)^36^.

Among the different fields of application, alkali-activation has been explored as a promising methodology for stabilizing bauxite, gold, copper, nickel, zinc, platinum, iron and sulphide-rich mine tailings, showing the positive influence of longer curing periods and temperature on strength development^12,37–43^.

Although studies are identified on mining tailings as precursor materials for alkaline activation and in situ applications, the stabilization of such materials still represents a field of research to be explored. Several questions required proper investigations, such as the identification of the main factors (e.g., dry unit weight, binder content, water content, and curing period) that influence the mechanical performance of the cemented materials, on the application of distinctive alkali-activated binders. In addition, an alkaline cement combining sugar cane bagasse ash and eggshell lime was never applied for mining tailings stabilization. Contributing to the advancement of this knowledge gap, this research evaluated the stabilization/solidification of iron mining tailings with an alkali-activated binder produced from sugar cane bagasse ash and hydrated eggshell lime. To this extent, unconfined compressive strength, split tensile strength, initial shear stiffness, and microstructural analyses were conducted. In addition, a rational dosage methodology was also applied.

## Materials and methods

### Materials

The materials applied in this research were iron ore tailings (IOTs); sugar cane bagasse ash (SCBA); hydrated eggshell lime (HEL) and sodium hydroxide (NaOH). IOTs were provided by an iron mining industry, located in the state of Minas Gerais (MG)—Brazil.

The SCBA was provided by a sugarcane processing industry, located in the state of Rio Grande do Sul (RS) Brazil. The IOTs were subjected to drying (50 °C for 48 h) and SCBA, drying and (50 °C for 48 h) sieving (200 mesh sieve, 75 µm opening). HEL was produced in the laboratory, from eggshells locally collected in Brazil. The production of HEL comprised the following processes: washing (i), drying (50 °C for 12 h) (ii), milling (iii) and calcinination (1050 °C for 4 h) (iv), hydration in distilled water (48 h) (v), and sieving (75 µm size) (vi)^44^. NaOH was acquired from a local company of chemical reagents in Brazil. The alkali-activated binder (AAB) was composed of a SCBA/HEL ratio of 80/20, with a molar concentration of activating solution (NaOH) of 1 M and respective concentration of alkalis (Na_2_O) of 2.61%. Further information on the binder production can be found in the work of Araújo et al.^45^.

### Characterization

**Figure 1:**
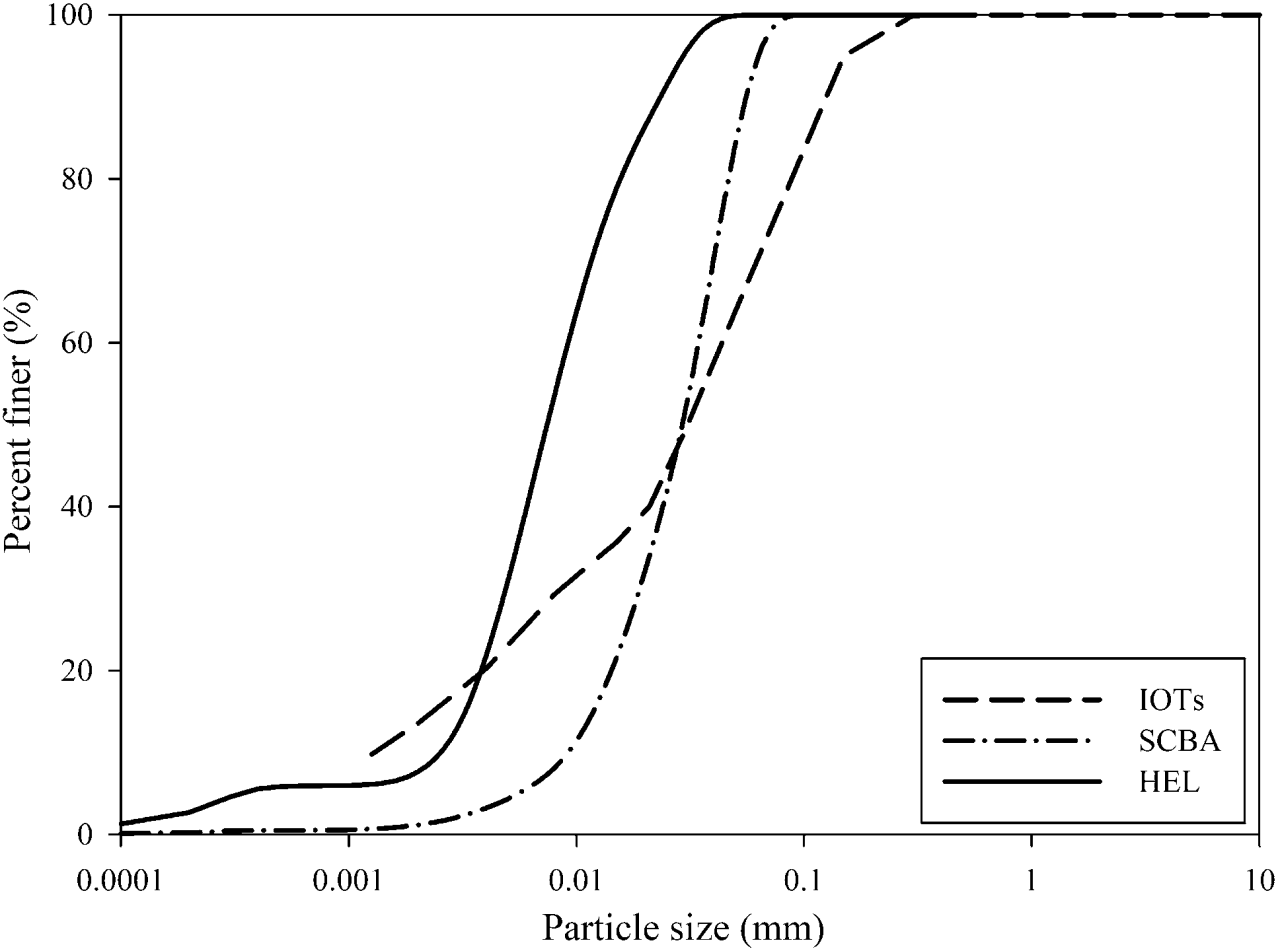
Grain size distribution of the materials.

**Figure 2:**
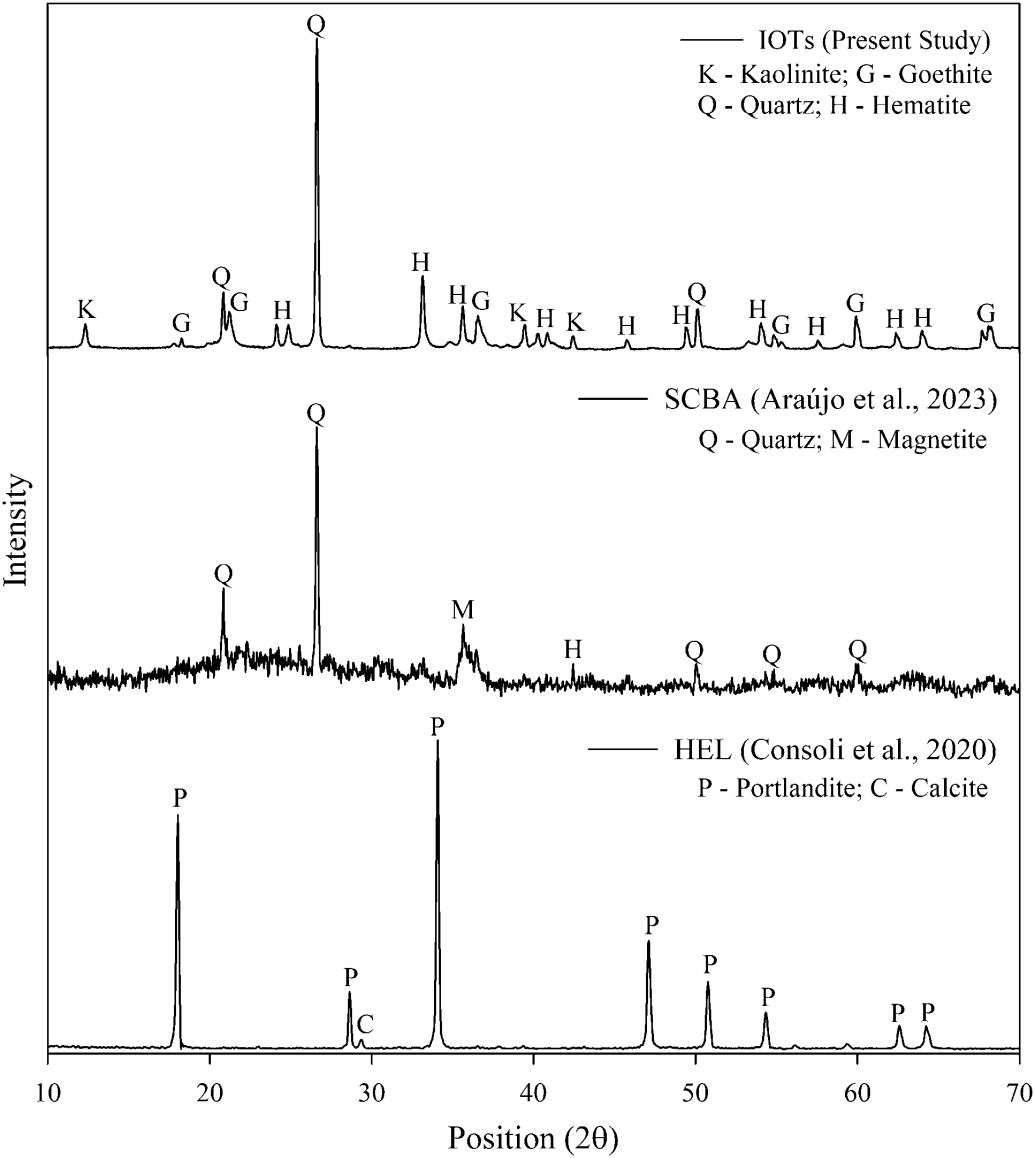
Mineralogical composition of IOTs, SCBA and HEL.

Materials characterization (Tables 1, 2, 3, Figs. 1, 2) was evaluated by determining their specific weight of grains [D854^46^; NBR 16605^47^]; specific surface area (Brunauer–Emmett–Teller method—BET); Atterberg limits [D4318^48^]; grain size distribution [D7928^49^; laser granulometry], and chemical (X-ray fluorescence spectrometry, XRF) and mineralogical (X-ray diffraction, XRD) composition. The grain size distribution of SCBA and HEL was determined using the laser diffraction technique, in a particle size analyzer (Cilas brand, model 1064). The specific surface area of the SCBA and HEL were measured by the BET method, using the QuantaChrome equipment (model NOVA 1200e).

**Table 1:** Materials physical properties.

Property	IOT	SCBA	HEL
Liquid limit (%)	–	–	–
Plasticity limit (%)	–	–	–
Plasticity index	NP	NP	NP
Specific gravity (g cm^−3^)	3.13	2.08	2.24
Surface area (m^2^ g^−1^)	–	125.15	4.18
Coarse sand (%)—0.6 ≤ d < 2 mm	0	0	0
Medium sand (%)—0.2 ≤ d < 0.6 mm	0	0	0
Fine sand (%)—0.06 ≤ d < 0.2 mm	48.95	8.88	0
Silt (%)—0.002 ≤ d < 0.06 mm	30.72	90.13	93.01
Clay (%)—d < 0.002 mm	20.33	0.99	6.99
USCS classification	ML	ML	ML

*NP* non-plastic, *ML* inorganic silt.

**Table 2:** Chemical composition of materials.

Oxides (%)	SiO_2_	Al_2_O_3_	Fe_2_O_3_	MnO	MgO	CaO	Na_2_O	K_2_O	TiO_2_	P_2_O_5_	Loss on ignition
IOTs	35.1	8.48	49.3	1.29	0.20	0.07	–	0.06	0.32	0.31	4.61
SCBA	60.65	5.76	13.87	0.45	1.97	1.40	0.22	2.90	4.14	1.26	7.38
HEL	0.12	0.13	0.15	–	1.17	72.9	–	–	–	–	25.14

**Table 3:** Factorial design unconfined compressive strength tests.

Controllable factors	Symbol	Coded factors		
		− 1	0	+ 1
Curing period (days)	A	7.0	17.5	28.0
Binder content (%)	B	15.0	20.0	25.0
Dry unit weight (kN m^−3^)	C	13.3	14.3	15.3
Water content (%)	D	14.6	18.7	22.8
Curing temperature (°C)	E	23.0	31.5	40.0

XRF was applied on molten samples (for major elements) and pressed samples (for minor elements), in the calibration of tabulated rock patterns, using an X-ray fluorescence spectrometer equipped with an Rh tube (Malvern Panalytical brand, model Zetiu). Loss on ignition (LOI) was performed at 1020 °C for 2 h. The XRD analyzes were carried out in an X-ray diffractometer [Siemens brand—BRUKER AXS, model D-5000 (θ–2θ)] equipped with a Cu fixed anode tube (λ = 1.5406 Å), operating at 40 kV and 25 mA. The powder samples were analyzed in the angular range of 2 to 72° 2θ in a step of 0.05°/1 s. The IOTs and the SCBA were evaluated for waste classification, according to NBR 10004^50^. The quantification of metals in the leached and solubilized extracts was carried out in an inductively coupled plasma atomic emission spectrometer (ICPE—brand Shimadzu, model ICPE-9800), using standard multi-element solution ICP Certipur (brand Merck).

All materials presented a non-plastic behavior and were classified as inorganic silts (ML) according to the Unified Soil Classification [D2487^51^]. As for the chemical composition, IOTs were mainly composed of iron oxides (49.3%), silicon (35.1%) and aluminum (8.48%) (Table 2). SCBA was mainly composed of silicon oxides (60.65%), iron (13.83%) and aluminum (5.76%)^38^, indicating a viable source of aluminosilicates. HEL corresponded to a rich source of calcium, equivalent to 72.9%^52^. The mineralogy of the IOTs consisted of kaolinite (Al_2_Si_2_O_5_(OH)_4_), goethite (Fe^3+^O(OH)), quartz (SiO_2_) and hematite (Fe_2_O_3_) (Fig. 2). SCBA presented semi-crystalline phases, with the presence of quartz and hematite^45^. The mineralogical composition of HEL comprises portlandite (Ca(OH)_2_) and calcite (CaCO_3_)^52^.

### Molding and curing procedures

**Figure 3:**
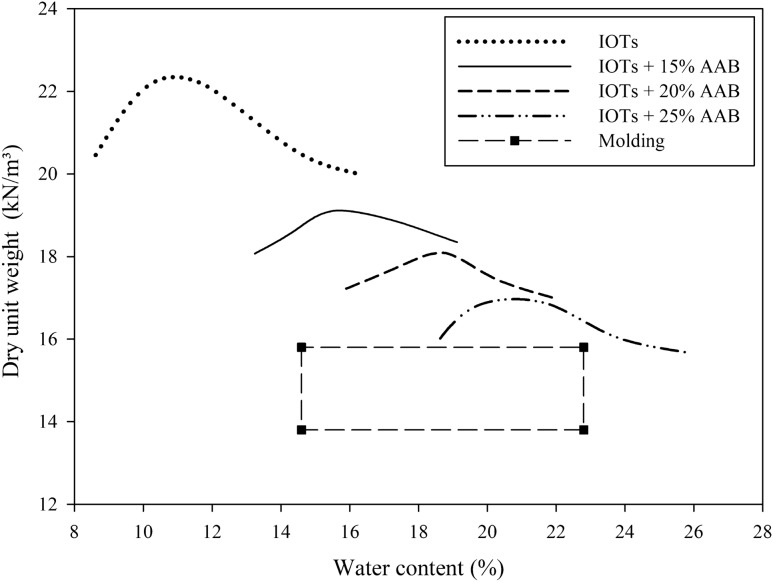
Compaction test IOT-ABB mixtures.

For IOTs stabilization/solidification, IOTs/AAB ratios of 85/15 and 75/25 were evaluated, based on previous studies regarding the stabilization of silt-like geotechnical materials with alkali-activated binders^26,38,41,53–57^. The dry unit weight and water content of the IOTs-AAB mixtures were defined in accordance with modified energy compaction test [D1557^58^], as shown in Fig. 3. To this extent, the dry unit weights (γ_d_) were defined as 13.3 kN m^−3^ and 15.3 kN m^−3^. Likewise, to explore the influence of the initial water content (w), distinctive points were defined in the dry-side and wet-side of the compaction curve, as follows: 14.6% (corresponding to a point below the optimal water content of the mixture with 15% AAB), and 22.8% (corresponding to a point above of the optimum water content of the mixture with 25% AAB).

For molding of the specimens, the mixture was statically compacted in three layers in a cylindrical specimen of 50 mm in diameter and 100 mm in height. After molding, the specimens were removed from the cylindrical molds and had their weights, diameters and heights measured with precision of 0.01 g and 0.1 mm, correspondingly. Afterwards, they were placed in hermetic bags, and kept at controlled temperature (23 ± 2 °C) and relative humidity (95% ± 2%) [D511^59^], during the predefined curing period. With 24 h remaining for the end of curing, specimens were immersed in water to reduce suction effects, as proposed by Consoli et al.^60^.

### Mechanical behavior tests

Unconfined compressive strength (UCS) and split tensile strength (STS) tests followed the procedures presented in ASTM C39^61^ and ASTM C496^62^. Both types of tests were conducted in an automatic press with a capacity of 100 tons (Engetotus brand) and rate of displacement of 1.14 mm per minute. To determine the initial stiffness (${G}_{0}$), interpreted as shear modulus at very small deformations, an ultrasonic pulse test was carried out with the aid of the PundiLab(+) instrument, in which measurements were made of the propagation times of the compression and shear waves by the cylindrical specimens. The compression wave was induced by the vibration of transducers at a frequency of 54 kHz, and the second (shearing) by the vibration of other types of transducers at 250 kHz.

### Design of experiments

To evaluate the unconfined compressive strength of the IOTs-AAB mixtures, a central composite factorial design with 5 factors and face-centered axial points (α = 1) was used. The combinations were performed in duplicate, with factorial (64), axial (20) and central (10) points, resulting in 94 experiments. This planning allowed evaluating the influence of controllable factors on the response variable, as well as mathematically modeling a response surface and identifying the existence of non-linearity^63^.

The following controllable factors were evaluated: curing time (A); binder content (B); dry unit weight (C); initial water content (D) and curing temperature (E). Curing periods of 7 and 28 days were adopted in order to evaluate the mechanical behavior over time. The AAB contents, as well as the study values for initial water content and specific weight were chosen from the compaction tests (Fig. 3). Curing temperatures of 23 °C and 40 °C were adopted considering: the first as the ambient temperature, and the second, as the maximum average temperature reached in southern Brazil^64^. The factors and their coded levels are shown in Table 3.

From the results obtained by the design of experiments carried out for the unconfined compressive strength tests, it was possible to create a reduced experimental planning for the split tensile strength and initial shear stiffness tests, with the curing temperature set at 23 °C and the initial water content at 22.8% (representing the best combination from UCS results), resulting in a simple factorial design composed of 3 factors. The combinations were performed in duplicate, resulting in 16 experiments, for the following controllable factors: curing time (A); binder content (B); specific weight (C). The factors and their coded levels are shown in Table 4.

**Table 4:** Factorial design split tensile strength and initial shear stiffness tests.

Controllable factors	Symbol	Coded factors	
		− 1	+ 1
Curing period (days)	A	7.0	28.0
Binder content (%)	B	15.0	25.0
Dry unit weight (kN m^−3^)	C	13.3	15.3

### Porosity/binder index (*η/B*_*iv*_)

The mechanical results were expressed as a function of the porosity/binder index proposed by Ref.^13^ and defined by Eqs. (1) and (2). Porosity (η) is a function of the dry unit weight (γd) and the contents of iron ore tailings (IOTs), sugarcane bagasse ash (SCBA) and hydrated eggshell lime (HEL). Each of these materials has a specific mass (γsIOT, γsSCBA, γsHEL), necessary for porosity determination (Eq. 1). The binder content (Biv) results from the division between the volume occupied by the SCBA and HEL and the total volume of the sample (Eq. 2).

1 $$\eta = 100 - 100\left\{ {\left[ {\frac{{\gamma_{d} }}{{\frac{IOTs}{{100}} + \frac{SCBA}{{100}} + \frac{HEL}{{100}}}}} \right]\left[ {\frac{{\frac{IOTs}{{100}}}}{{\gamma_{{s_{IOTs} }} }} + \frac{{\frac{SCBA}{{100}}}}{{\gamma_{{s_{SCBA} }} }} + \frac{{\frac{HEL}{{100}}}}{{\gamma_{{s_{HEL} }} }}} \right]} \right\},$$

2 $${B}_{iv}=\frac{{V}_{SCBA}+{V}_{HEL}}{V}=\frac{\frac{{m}_{SCBA}}{{\gamma }_{{s}_{SCBA}}}+\frac{{m}_{HEL}}{{\gamma }_{{s}_{HEL}}}}{V}.$$

This index allows the unification of all experiments in a single relation, resulting in a rational dosage methodology for any cemented soil mixture, replacing trial and error conventional strategies that are laborious and time consuming. Nevertheless, the proposed equations are only valid for the cemented mixtures studied herein and functional if the boundary conditions of the applied variables are ensured.

### Chemical and mineralogical analyses

The mineralogy of the mixtures with the best mechanical behavior, for different temperatures and curing times, were evaluated using XRD and FTIR (Fourier Transform Infrared) techniques. The XRD analysis of the mixtures was performed with technical specifications equal to those described in the item “Characterization”. The FTIR spectra analyzed Chemical bonds and was conducted in a Perkin Elmer FTIR Spectrometer (model Spectrum 1000) within the range of 4000–40,000 cm^−1^ and at a resolution of 4 cm^−1^.

### Microstructural evaluation

The microstructure of the mixtures with the best mechanical behavior, for different temperatures and curing times, were evaluated using SEM (scanning electron microscopy) technique. The SEM of the mixtures was performed in a scanning electron microscope (Tescan brand, model Vega 3), using the SE (Secondary electrons) with magnification of 10,000 times, electron beam of 20 kV, and gold-coated samples.

## Results and discussion

### Unconfined compressive strength

**Figure 4:**
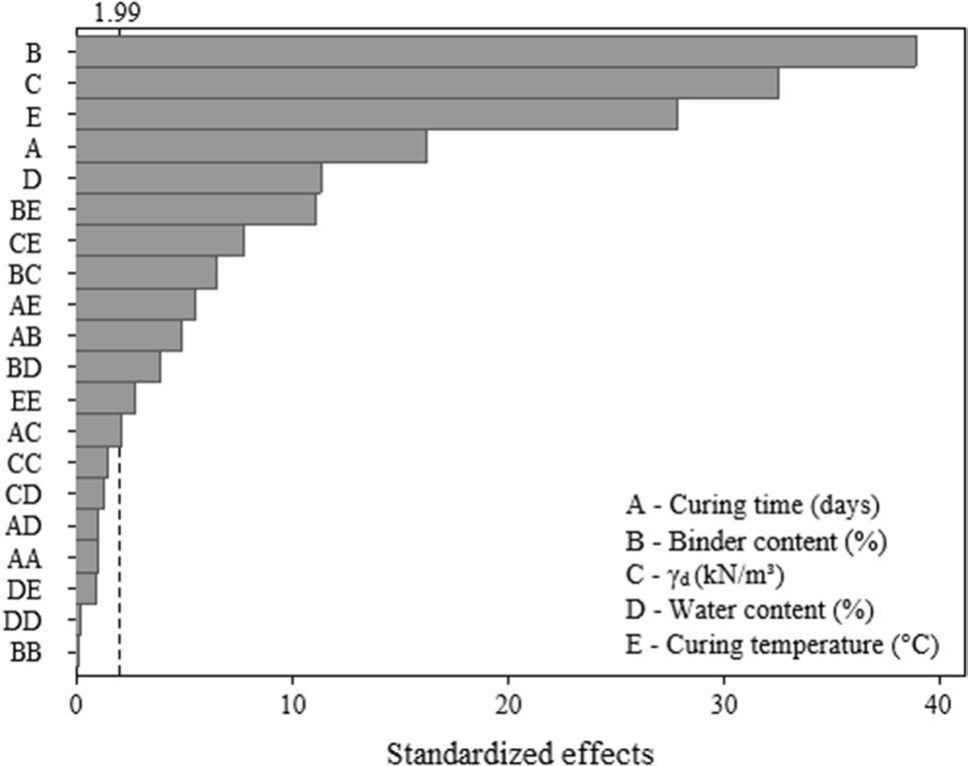
Pareto chart of ${q}_{u}$ for IOT-AAB.

**Figure 5:**
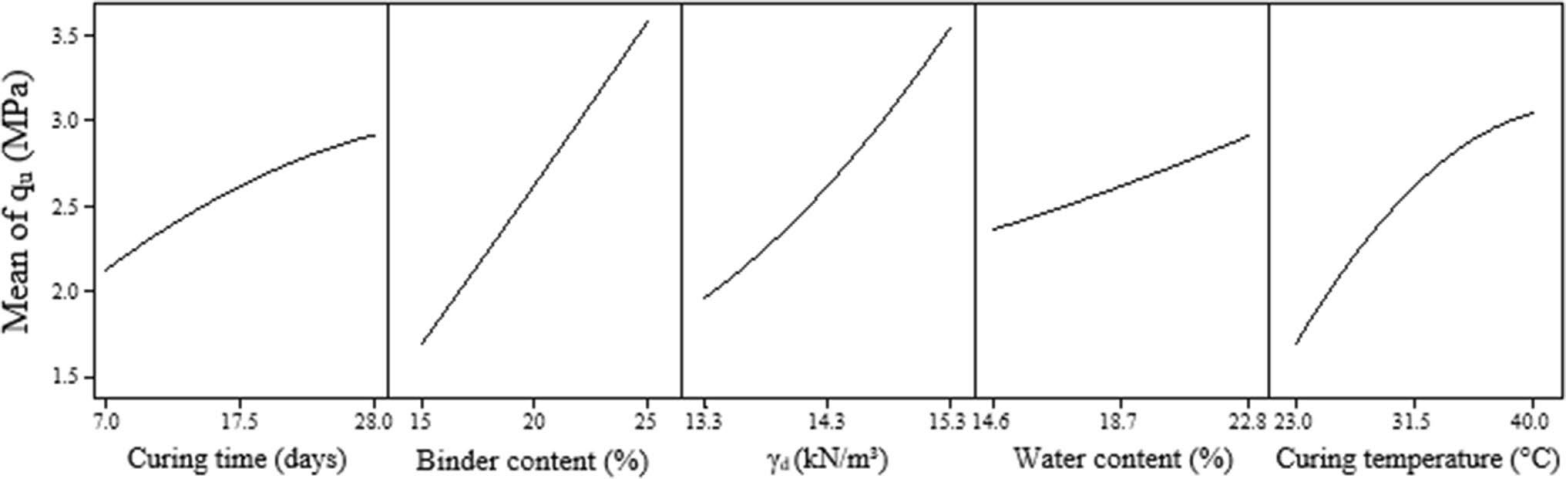
Main effects plot of ${q}_{u}$ for IOTs-AAB.

Figure 4 presents the evaluation of the significance of the controllable factors (represented by letters A, B, C, D, and E), and the interactions between these factors (represented by combinations of letters) over the unconfined compressive strength (${q}_{u}$). It is noted that the ${q}_{u}$ of the IOTs-AAB mixtures is significantly influenced (p-value < 0.05) by all controllable factors and several second-order interactions (e.g. BE, CE, and BC). Binder content (B) and dry unit weight (C) are the factors that exert the greatest influence on ${q}_{u}$, followed by curing temperature (E), curing time (A) and initial water content (D). In the main effects plot (Fig. 5), all the controllable factors significantly influence in a positive way the ${q}_{u}$ of the IOT-AAB mixtures. In other words, the increase of the factor level results in the maximization of the strength. In addition, except for the curing temperature factor, all factors exert a linear influence on ${q}_{u}$.

The curing time factor (A) positively influences the quality of IOTs-AAB mixtures, in which cementitious products continue to form over time, contributing to strength development. Similar results were also observed in soil stabilization/solidification works with different alkali-activated binders^38,40^. The increase in the binder content factor (B) promoted the precipitation of a greater amount of cementing products, directly contributing to the maximization of ${q}_{u}$. In this system, the development of cementing reactions is intensified, due to the alkaline activation process, the binder content exerts greater influence when compared to the dry unit weight (Fig. 4). This observation is corroborated by results of work on stabilization of mining tailings using alkali-activated binders^38,40,41^. Higher levels on the dry unit weight factor (C) promoted greater contact points between the particles of the IOTs-AAB mixtures, due to the lower porosity, enhancing a greater mobilization of friction and interlocking, directly contributing to the increase in strength.

In alkali-activated systems, such as IOTs-AAB, the initial water content (D) acts as a mean for the condensation-polymerization reactions to occur, requiring a minimum amount of water for the formation of cementing products, ranging according to the precursors and activators used in the process^65,66^. In this work, SCBA is a material that has a high specific surface area (Table 1), with a high water absorption capacity. Thus, the initial water content variation from 14.6 to 22.8% was an important factor for the alkaline activation process in the IOTs-AAB system. The increase in curing temperature (E), from 23 to 40 °C, acted as a catalyst for the dissolution reaction of aluminosilicates, accelerating the formation of cementing gels and making it possible to obtain greater strength in a shorter period of time. Bhagath Singh and Subramaniam^67^ report similar results with alkali-activated cementing binders, in which the increase in the rate of the dissolution process and consequent improvement in the mechanical behavior was verified with the increase in the curing temperature (from 25 to 40 °C).

**Figure 6:**
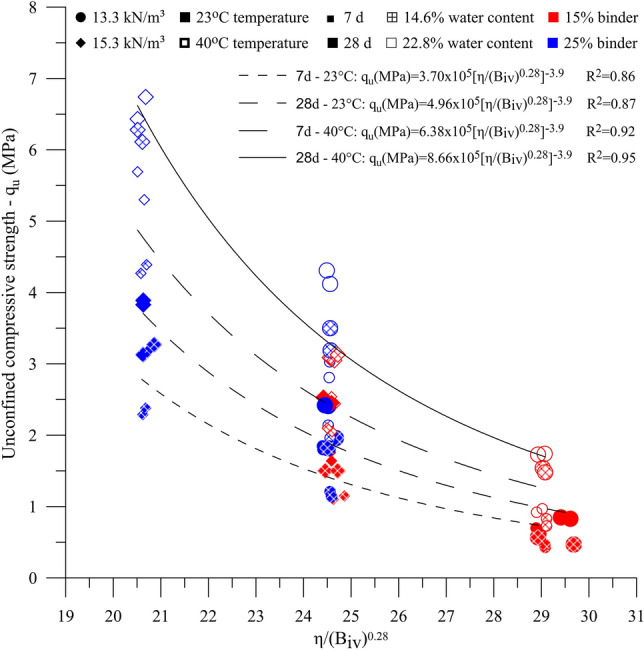
${q}_{u}$ versus η/${B}_{iv}$ for IOT-AAB.

The ${q}_{u}$ results were also correlated with the η/B_iv_ index, as shown in Fig. 6. In general, the IOTs-AAB treatments cured for 7 days at 40 °C showed average strength higher than specimens cured for 7 days at 23 °C and specimens cured for 28 days at 23 °C. The best IOTs-AAB treatment (25% AAB, 22.8% water content and 15.3 kN m^−3^ dry unit weight) reached an average ${q}_{u}$ of 6.59 MPa for 28 days curing at 40 °C, representing an increase of 110% compared to 7 days curing at 23 °C specimens (3.14 MPa).

An exponent of 0.28 was applied to the ${B}_{iv}$ parameter (Fig. 6). This exponent was chosen considering that it represents the best adjustment to the results of unconfined compressive strength, in line with studies on the stabilization of mining tailings with alkali-activated binders^38–40^. The relationships between the experimental data and the η/B_iv_^0.28^ index showed good coefficients of determination (R^2^), ranging from 0.86 to 0.95. These coefficients suggest the feasibility of the index for predicting the ${q}_{u}$ of the IOTs-AAB mixtures. The reduction in the η/B_iv_^0.28^ index led to an increase in strength; through this index it becomes possible to choose the most appropriate solution to achieve the strengths of interest: reducing porosity (increasing the compaction of the mixture) or increasing the binder content for IOT stabilization/solidification. Considering that the DNIT 143 standard^68^ requires a minimum unconfined compressive strength of 2.1 MPa for soil–cement pavement bases cured for 7 days at 23 °C, a η/B_iv_^0.28^ of 22.1 (Eq. 7d—23 °C, Fig. 6) for IOT-AAB mixtures would be necessary to fulfill this requirement.

### Split tensile strength

**Figure 7:**
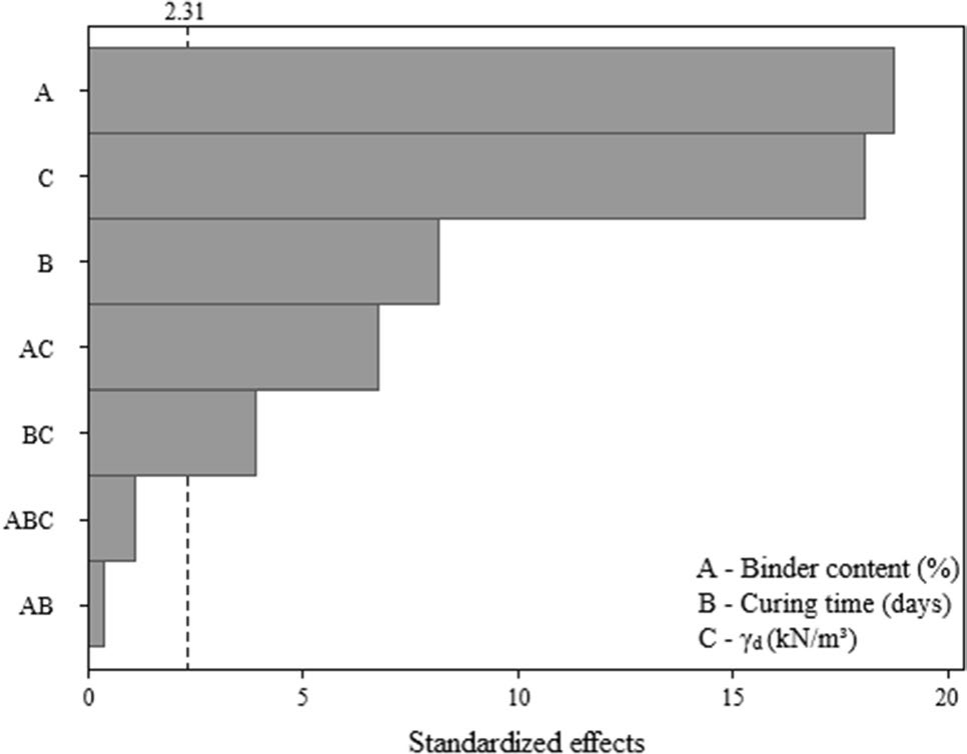
Pareto chart of ${q}_{t}$ for IOTs-AAB.

**Figure 8:**
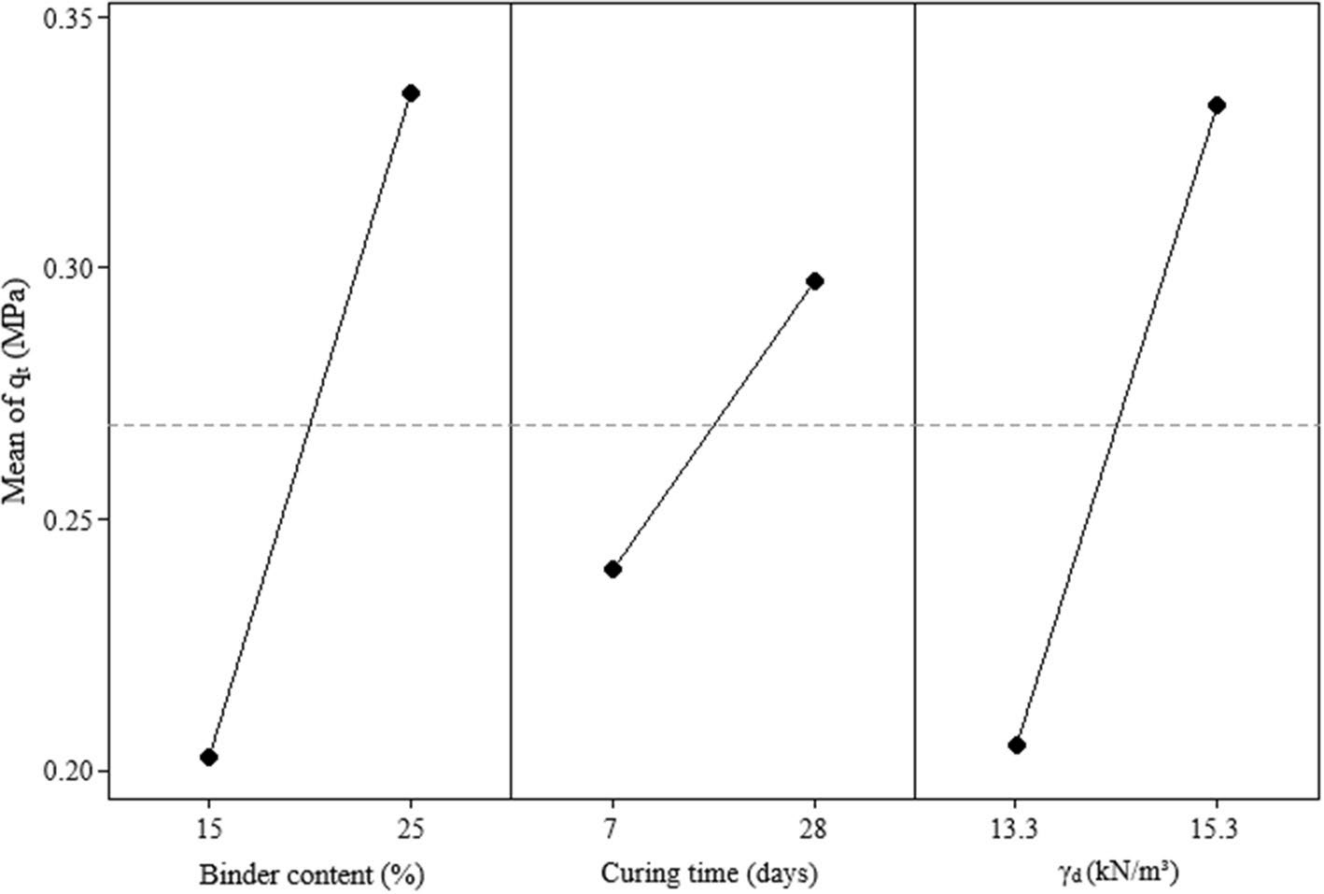
Main effects plot of ${q}_{t}$ for IOTs-AAB.

Similar to unconfined compressive strength results, the split tensile strength of the IOTs-AAB mixtures seems (Fig. 7) to be more significantly influence by binder content (A), followed by dry unit weight (C) and curing period (B), as well as second order interactions (AC and BC). Along these lines, Fig. 8 depicts the main effects plot, showing that the increase in all main factors positively influence the response variable (i.e., split tensile strength).

**Figure 9:**
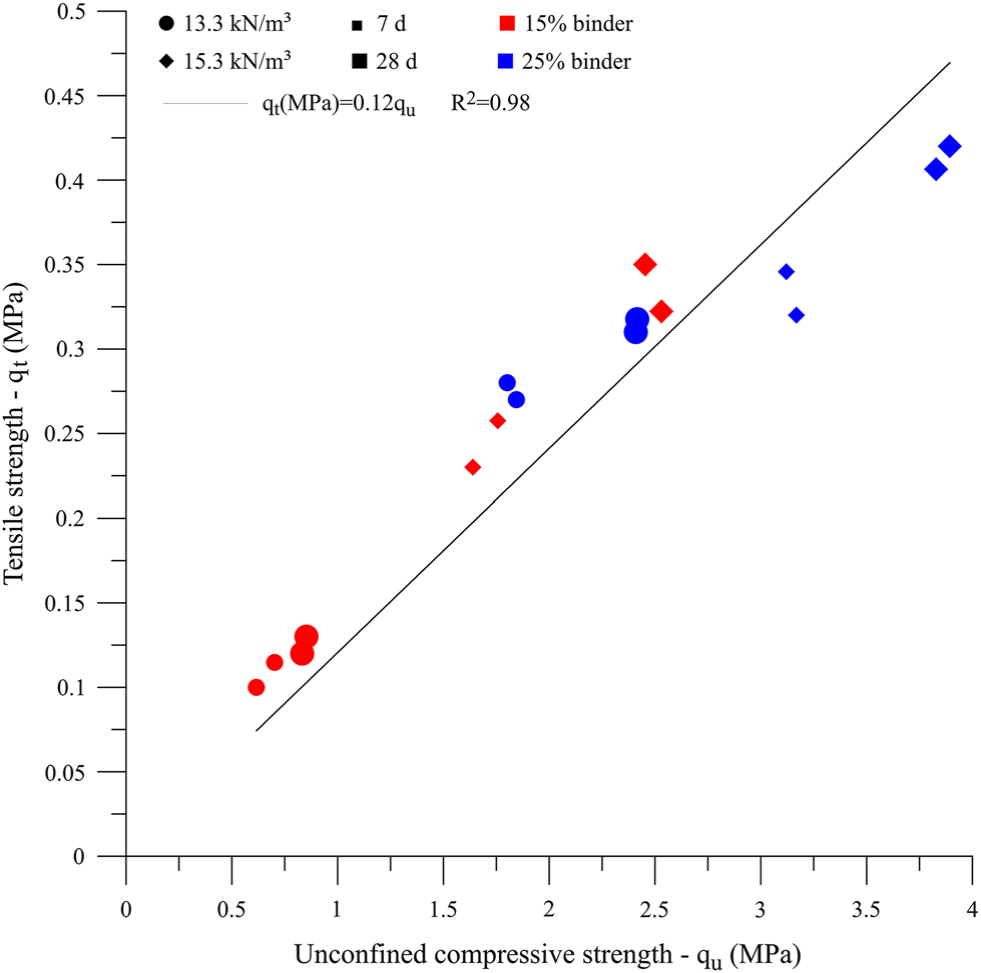
${q}_{u}$ versus ${q}_{t}$ for IOT-AAB mixtures.

Higher ${q}_{t}$ values were achieved in IOTs-AAB mixtures with higher binder content, dry unit weight and curing time, reaching an average strength of 0.42 MPa after 28 days at 23 °C. The explanations behind the influence of the aforementioned factors on the ${q}_{t}$ are the same as the ones presented for ${q}_{u}$. This statement is supported by the data shown in Fig. 9, in which a linear correlation was obtained between ${q}_{u}$ and ${q}_{t}$, with R^2^ of 0.98. The strength ratio (${q}_{t}$/${q}_{u}$) was 0.12, i.e., ${q}_{t}$ corresponds to 12% of the ${q}_{u}$ for IOT-AAB mixtures. This value is aligned with previous values found in the literature for cemented soils^29,45^.

**Figure 10:**
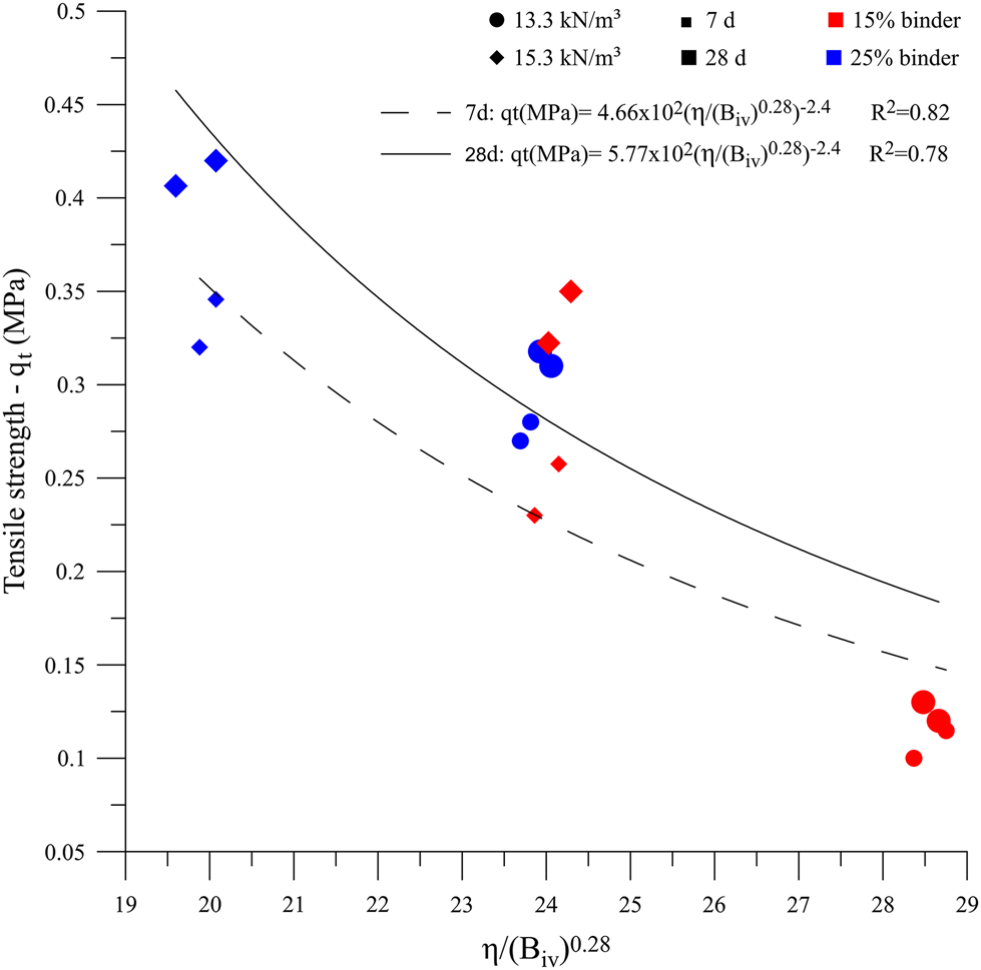
${q}_{t}$ versus η/${B}_{iv}$ for IOT-AAB.

Figure 10 shows that the reduction of the porosity/binder index resulted in higher ${q}_{t}$. The AAB equations that relate the experimental data and the η/B_iv_^0.28^ index showed coefficients of determination of 0.82 and 0.78, indicating the viability of using the index to predict the ${q}_{t}$ for IOT-AAB.

### Initial shear stiffness

**Figure 11:**
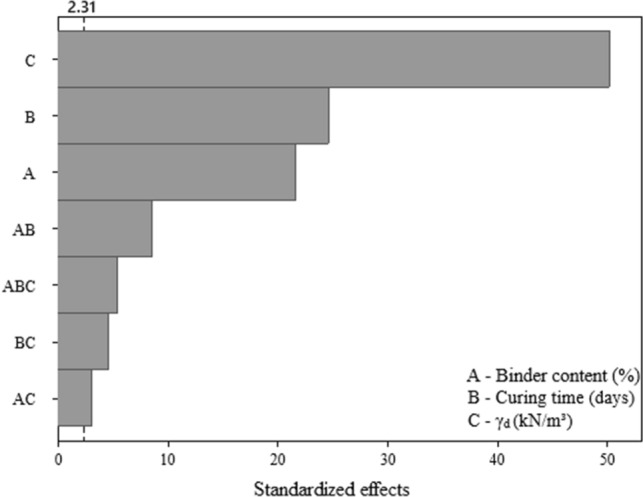
Pareto chart of ${G}_{0}$ for IOTs-AAB.

**Figure 12:**
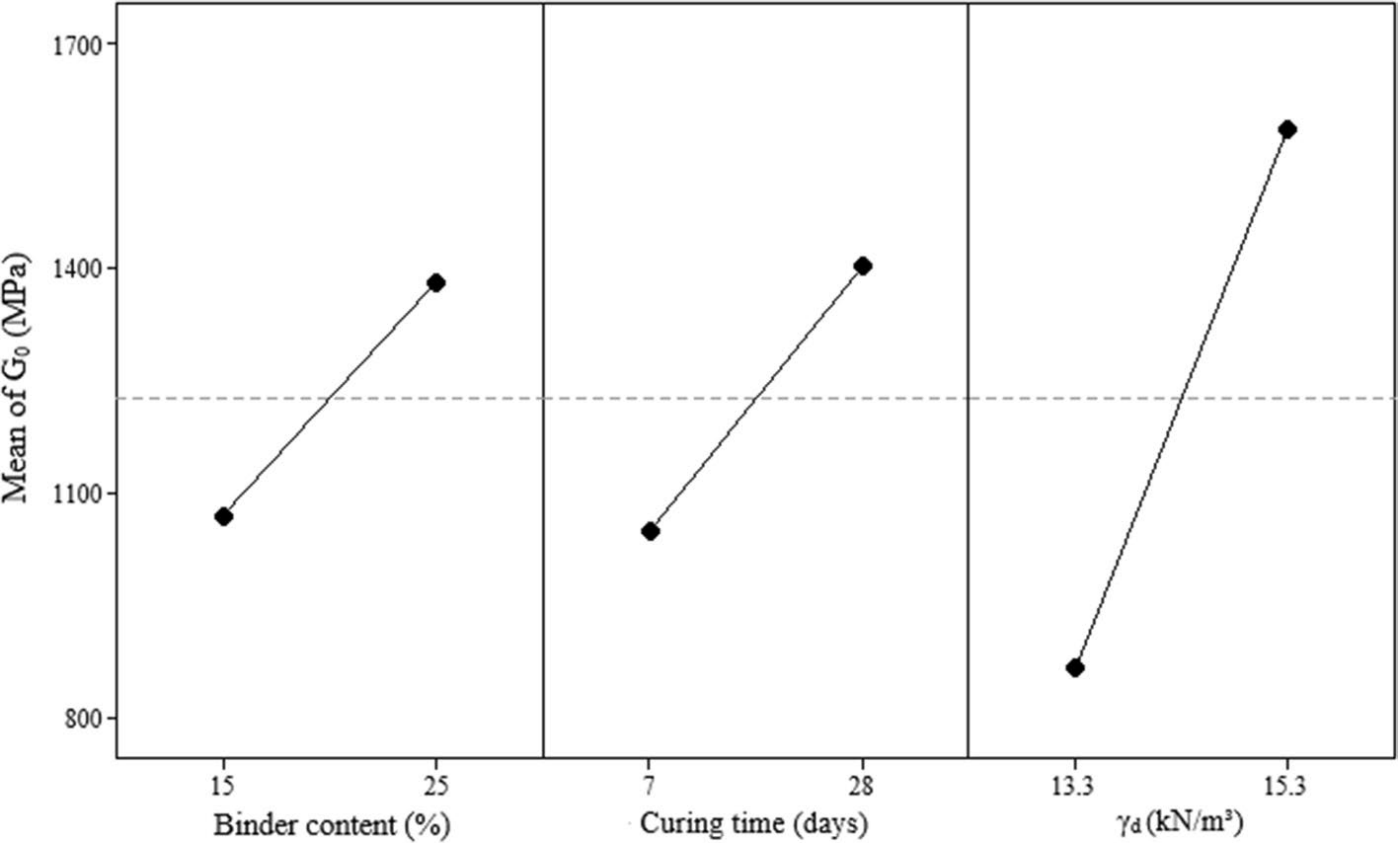
Main effects plot of ${G}_{0}$ for IOTs-AAB.

**Figure 13:**
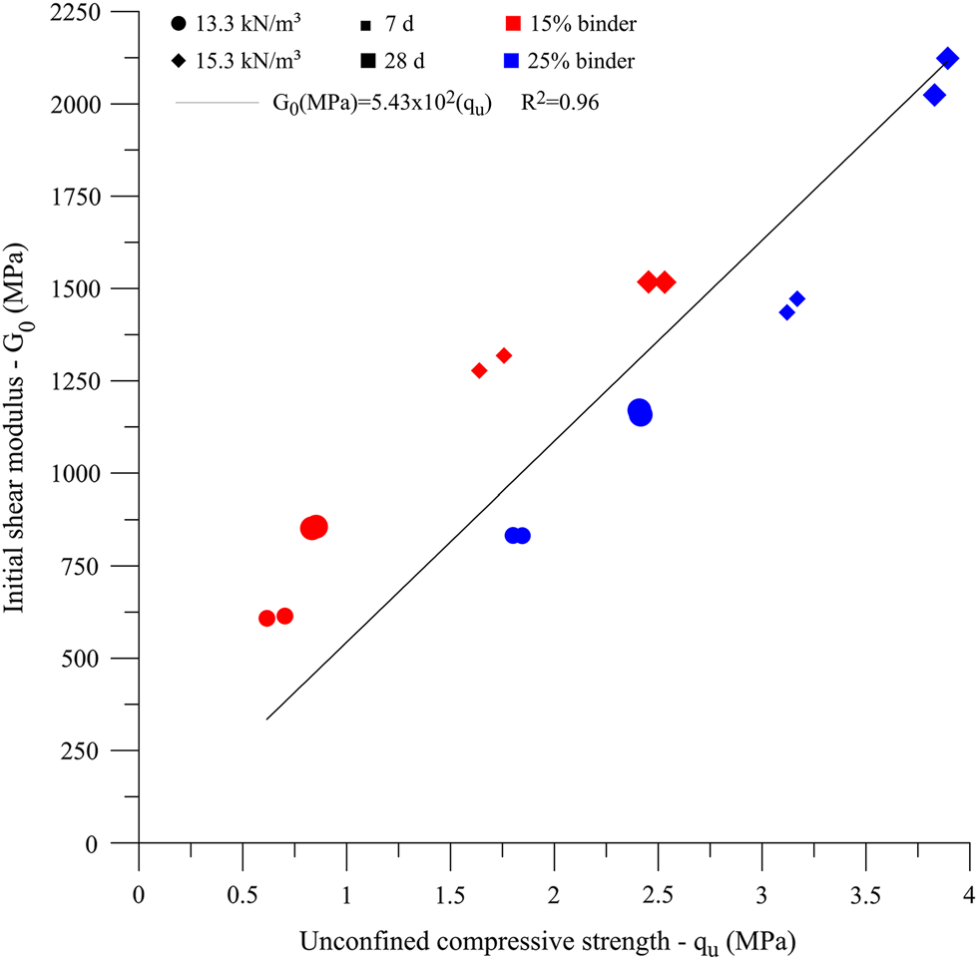
${q}_{u}$ versus G_0_ for IOT-AAB mixtures.

The Pareto chart (Fig. 11) for initial shear stiffness results shows that all controllable factors significantly influence the response variable. Regarding the magnitude of this influence, the following order (from higher to lower) was observed: dry unit weight (C), curing period (B), and binder content (A). The main effect plot of initial shear stiffness (Fig. 12) also indicates that the increase in all main factors leads to an improvement on the response variable, such as the case of unconfined compressive strength and split tensile strength results. The influence of controlled factors on the ${G}_{0}$ depicts a qualitatively similar trend as for *q*_*u*_ of the IOTs-AAB mixtures; this statement is supported by Fig. 13, in which a linear correlation was obtained between *q*_*u*_ and ${G}_{0}$, with R^2^ of 0.96. Thus, it is expected that higher unconfined compressive strength values will be reached by IOTs-AAB mixtures that are initially stiffer. This correlation between mechanical responses (*q*_*u*_ and ${G}_{0}$) was also observed in other studies on soil stabilization with alkali-activated binders^26,69^.

**Figure 14:**
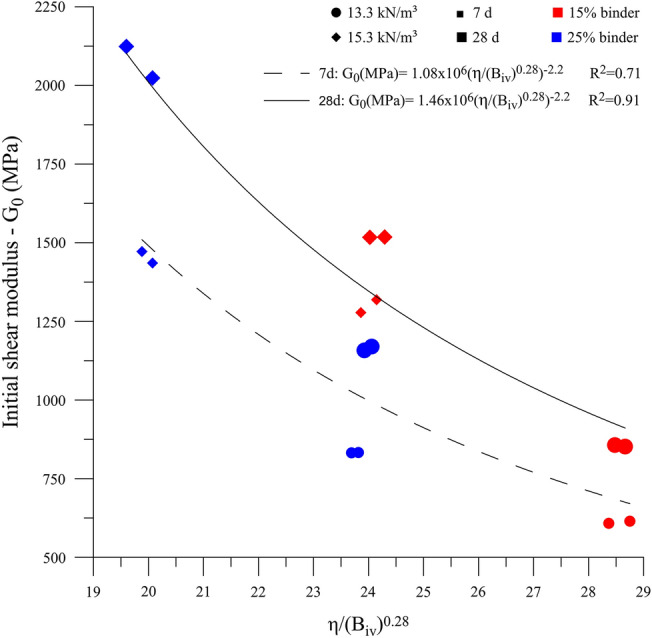
${G}_{0}$ versus η/${B}_{iv}$ for IOT-AAB.

Figure 14 shows that the reduction in the porosity/binder content ratio led to higher values of ${G}_{0}$, reaching an average stiffness of 2073.7 MPa after 28 days at 23 °C. This behavior was also observed in clayey soil^45^ and mining tailings^38,40^ stabilized with alkaline cements. The relationships between experimental data and η/B_iv_^0.28^ showed satisfactory R^2^ values (0.71 and 0.91), indicating that the porosity/binder index is an adequate parameter for predicting the initial stiffness of IOTs-AAB mixtures.

Regarding the mechanical responses, ${q}_{u}$, ${q}_{t}$ and ${G}_{0}$ of the IOT-AAB mixtures are significantly influenced by all controllable factors, mainly by binder content and dry unit weight. Furthermore, the curing temperature has an important influence on the strength of the cemented material. The results of strengths and initial stiffness showed that IOT-AAB presents mechanical behavior considered satisfactory.

### Mineralogy

**Figure 15:**
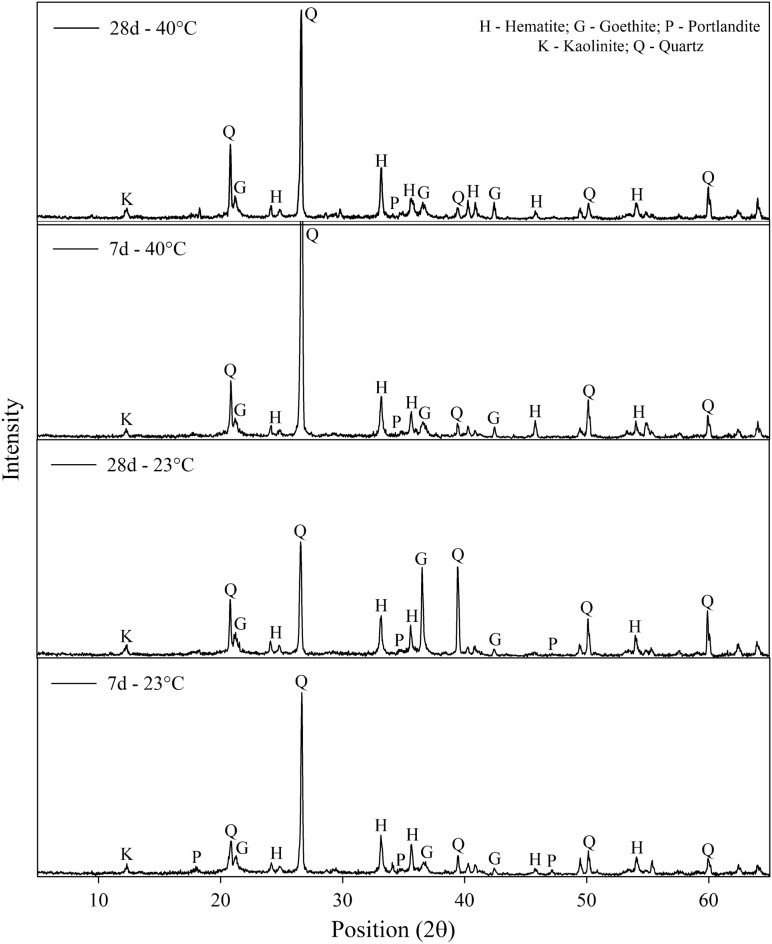
Diffractograms of IOTs-AAB mixtures.

Figure 15 shows the diffractograms of the IOTs-AAB mixture with the best mechanical behavior (25% binder content, 22.8% water content and 15.3 kN m^−3^ dry unit weight), for different curing periods (7 and 28 days) and temperatures (23 and 40 °C). The samples present a mineralogy composed of semi-crystalline and crystalline phases, which share the presence of kaolinite (Al_2_Si_2_O_5_(OH)_4_) and goethite (Fe^3+^O(OH) (present in the IOTs), quartz (SiO_2_) and hematite (Fe_2_O_3_) (from IOTs and SCBA) and portlandite (Ca(OH)_2_) (from HEL). It is also observed that portlandite is being consumed due to interactions with aluminosilicates, over time (7 to 28 days) and curing temperature (23 to 40 °C), forming a cementitious product, which corroborates the influence of these factors observed on the mechanical behavior of the IOTs-AAB mixtures. In all samples, the presence of amorphous phases was detected, which corresponds to N-A-S-H gel within the 2θ angular range of 20°–35°^70^. XRD analysis provided qualitative information that was later confirmed using FTIR.

**Figure 16:**
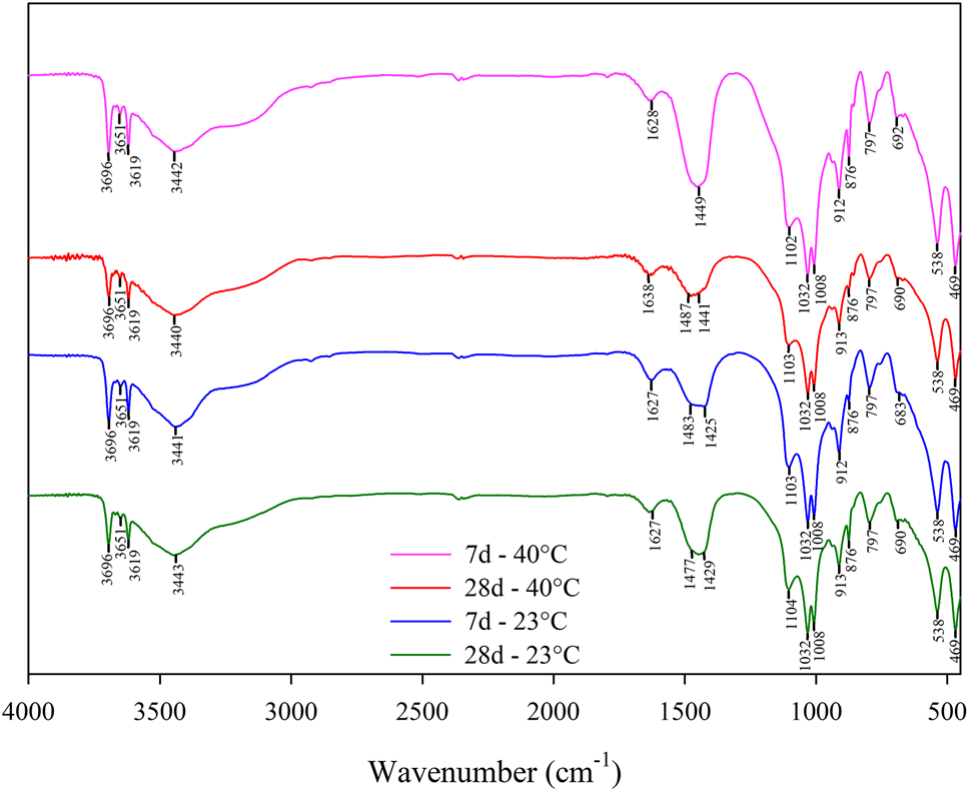
FTIR spectra of IOTs-AAB mixtures.

From the FTIR analysis (Fig. 16) it was possible to evaluate the chemical compounds present in the IOTs-AAB mixtures, further corroborating the qualitative information provided by the XRD results. Peaks 3619 and 3696 cm^−1^ are observed for both samples. According to Ref.^71^, this band range is attributed to the O–H stretching linked to the residual modes of kaolinite, goethite, axial asymmetric and symmetric Al–O–H and tetrahedral Al. The 3651 cm^−1^ band corresponds to portlandite^72^ corroborating the verification of the mineral in the diffractograms of the IOTs-AAB mixtures (Fig. 15). The presence of this mineral indicates that there is still portlandite to be consumed in the cementing gel formation reactions. The bands between 3440 and 3443 cm^−1^ are associated with stretching vibrations of water molecules (H–O–H bond). The region of the absorption band between 1627 and 1638 cm^−1^ indicates the H–O–H bending vibration^73^.

The absorption band between peaks 1002 and 1004 cm^−1^ may be related to asymmetric stretching vibrations of Si–O bands present in the samples^74^. The bands at 1008, 797 and 469 cm^−1^ are related to the elongation vibration of Fe–O–H bonds attributed to goethite and in the same sense, the lower elongation band at 538 cm^−1^ is associated with the presence of hematite with identical bonds^28,72^. The elongation in the 876 cm^−1^ band can represent a Si–O–H bending mode or Al tetrahedral bending^75^ both elements that make up the majority of the base materials ratio of the IOT-AAB mixture. The bands between 683 and 692 cm^−1^ show characteristics of symmetrical vibration of quartz Si–O–T bonds^28,74^. The bands around 1425–1487 cm^−1^ refer to the elongation of the C–O bonds, resulting in calcined and untreated geopolymer specimens due to the inevitable formation of carbonate from the reaction of alkali metal oxide (NaOH) with the ambient air^71,76^. The band at 1032 cm^−1^ (present in all samples) corresponds to asymmetric stretching vibrations of Si–O–T (T = Al, Fe or Si), characteristics of the presence of N-A-S-H gel^71,76^.

### Microstructure

**Figure 17:**
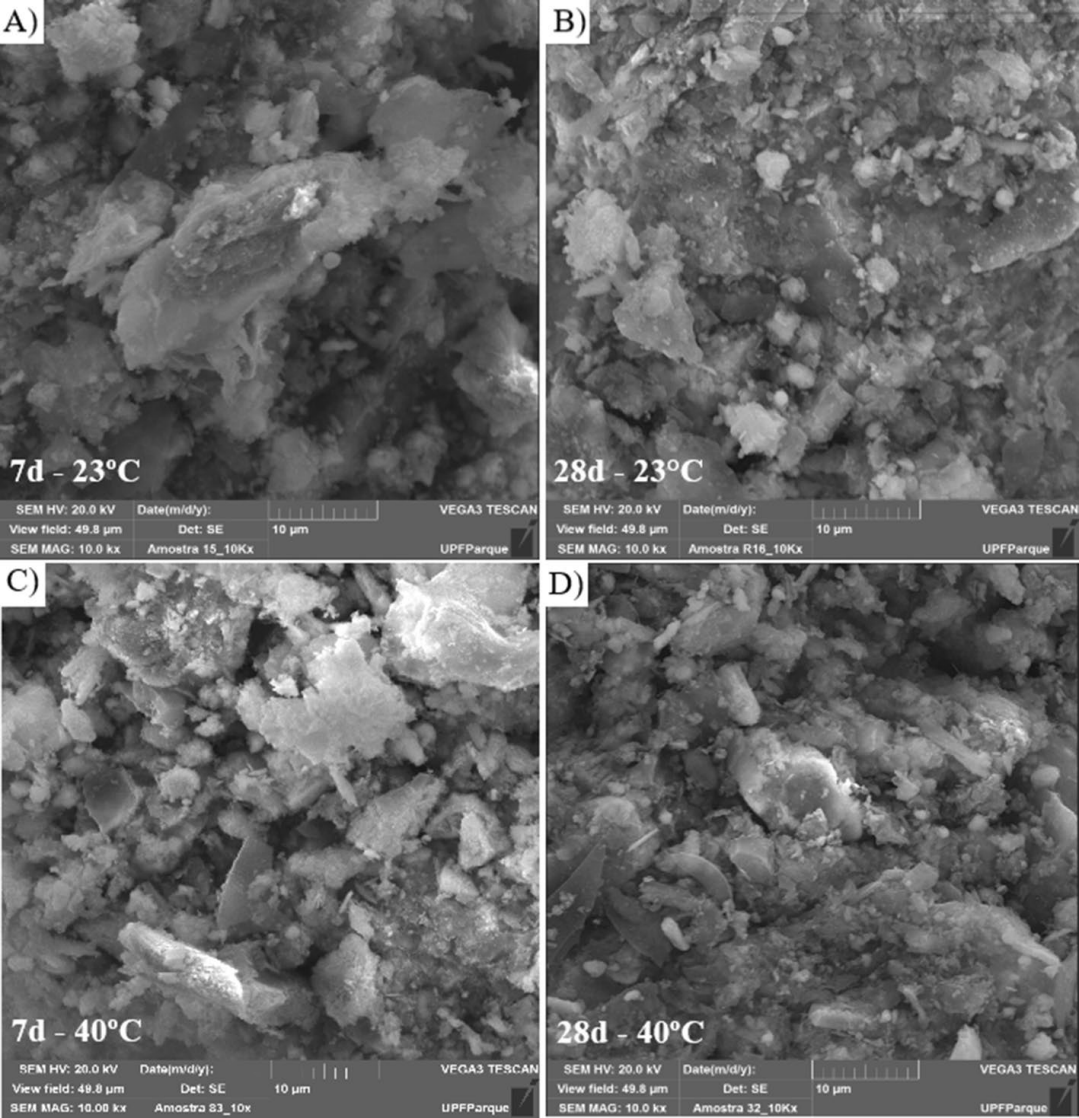
SEM images of IOTs-AAB (10 kx magnification): (**a**) 7 days—23 °C, (**b**) 28 days—23 °C, (**c**) 7 days—40 °C, (**d**) 28 days—40 °C.

From the SEM images (Fig. 17), the mixture with the lowest temperature and curing time (Fig. 17a) showed a greater ratio of points containing voids between the particles in relation to the other IOTs-AAB mixtures (Fig. 17b–d). This observation can be related to the chemical reactions of the formed cementing gel (N-A-S-H gel) from the AAB with the IOTs particles under high temperatures and curing times, thus filling the voids, and corroborating the increase in strength observed in the mechanical results (Fig. 6).

The results of microstructural analyses (XRD, FTIR and SEM) revealed the formation of the N-A-S-H gel and its distribution in the cemented matrix. These findings justify the increases in strengths and initial stiffness of the IOT-AAB mixtures, demonstrating the efficiency of the alkaline cement in stabilizing/solidifying mining tailings.

## Conclusions

From the tests and analyzes carried out in this research, the following final considerations are presented:

- The unconfined compressive strength, split tensile strength, and initial shear stiffness of the IOTs-AAB mixtures were significantly influenced by all the evaluated factors, mainly by binder content and dry unit weight. Furthermore, increasing the curing temperature from 23 to 40 °C resulted in the maximization of unconfined compressive strength due to the acceleration of chemical reactions;
- Mixtures with higher unconfined strength also presented in higher split tensile strength and initial shear stiffness, indicating a direct correlation between strength and stiffness for all studied combinations;
- η/B_iv_^0.28^ index proved to be an adequate parameter to evaluate the stabilization/solidification of IOTs regarding unconfined compressive strength, split tensile strength, and initial shear stiffness. This index allowed the unification of the results in a single relation, replacing trial and error conventional strategies that normally are laborious and time-consuming;
- The microstructural analysis showed that increasing the temperature and curing time of IOTs-AAB mixtures, increases the consumption of portlandite from HEL, enhancing the formation of the cementing gel (N-A-S-H). The increase in curing period also led to a reduction in the voids of the mixtures, corroborating the behavior observed in the mechanical results.

## Data Availability

The datasets used and/or analyzed during the current study are available from the corresponding author on reasonable request.
